# Individuals’ expected genetic contributions to future generations, reproductive value, and short‐term metrics of fitness in free‐living song sparrows (*Melospiza melodia*)

**DOI:** 10.1002/evl3.118

**Published:** 2019-04-25

**Authors:** Jane M. Reid, Pirmin Nietlisbach, Matthew E. Wolak, Lukas F. Keller, Peter Arcese

**Affiliations:** ^1^ School of Biological Sciences University of Aberdeen Aberdeen United Kingdom; ^2^ Department of Zoology University of British Columbia Vancouver British Columbia Canada; ^3^ Department of Biological Sciences Auburn University Auburn Alabama 36849; ^4^ Department of Evolutionary Biology & Environmental Studies University of Zurich Zurich Switzerland; ^5^ Forest & Conservation Sciences University of British Columbia Vancouver British Columbia Canada

**Keywords:** Evolutionary dynamics, fitness, genealogy, gene‐dropping, individual reproductive value, lifespan, lifetime reproductive success, pedigree, population growth rate

## Abstract

Appropriately defining and enumerating “fitness” is fundamental to explaining and predicting evolutionary dynamics. Yet, general theoretical concepts of fitness are often hard to translate into quantities that can be measured in wild populations experiencing complex environmental, demographic, genetic, and selective variation. Although the “fittest” entities might be widely understood to be those that ultimately leave most descendants at some future time, such long‐term legacies can rarely be measured, impeding evaluation of the degree to which tractable short‐term metrics of individual fitness could potentially serve as useful direct proxies. One opportunity for conceptual and empirical convergence stems from the principle of individual reproductive value (*V*_i_), here defined as the number of copies of each of an individual's alleles that is expected to be present in future generations given the individual's realized pedigree of descendants. As *V*_i_ tightly predicts an individual's longer term genetic contribution, quantifying *V*_i_ provides a tractable route to quantifying what, to date, has been an abstract theoretical fitness concept. We used complete pedigree data from free‐living song sparrows (*Melospiza melodia*) to demonstrate that individuals’ expected genetic contributions stabilize within an observed 20‐year (i.e. approximately eight generation) time period, allowing estimation of individual *V*_i_. Considerable among‐individual variation in *V*_i_ was evident in both sexes. Standard metrics of individual lifetime fitness, comprising lifespan, lifetime reproductive success, and projected growth rate, typically explained less than half the variation. We thereby elucidate the degree to which fitness metrics observed on individuals concur with measures of longer term genetic contributions and consider the degree to which analyses of pedigree structure could provide useful complementary insights into evolutionary outcomes.

**Impact Summary**

The concept of “fitness” is central to understanding how the frequencies of different genes within a population change over generations, and hence to understanding and predicting the progress of evolution. Many biologists would broadly agree with the overarching idea that the “fittest” genes, or individual organisms, are ultimately those that contribute the most descendant gene copies to a population at some point in the future. Yet long‐term genetic contributions are very difficult to measure, especially within the typical timeframes of research projects on free‐living populations. Consequently, biologists have rarely directly evaluated individuals’ longer term genetic contributions, or then evaluated the degree to which an individual's own reproductive output, which is somewhat easier to measure, adequately predicts its longer term contribution and hence could provide a reliable proxy indicator.

We used >20 years of unusually complete and accurate family‐tree (i.e., “pedigree”) data from free‐living song sparrows (*Melospiza melodia*) to quantify individuals’ genetic contributions to the focal population across multiple generations of descendants. We show that individuals’ expected contributions stabilized to an approximately constant value within the observed 20‐year timeframe, allowing individuals’ longer term genetic contributions to be inferred. These contributions varied substantially among individuals of each sex: some individuals left considerable genetic legacies in future generations, whereas other individuals left none. However, these longer term contributions were only partly predicted by an individual's own observed reproductive output, largely because some individuals that produced multiple offspring still had zero local genetic legacy in the longer term.

Our analyses illuminate the degree to which short‐term measures of individual reproduction recorded in wild populations experiencing naturally complex environmental and genetic variation can be used to directly infer longer term evolutionary outcomes. They also illustrate how analyses of individuals’ pedigrees could potentially provide additional insights into evolutionary outcomes that complement other approaches to evolutionary analysis.

Appropriately defining and enumerating “fitness” is fundamental to all theoretical and empirical attempts to explain and predict the dynamics of allele frequencies, phenotypes, and populations (de Jong 1994; Day and Otto 2001; Grafen 2006; Orr 2009; Sæther and Engen 2015). Yet it has proved hard to define quantitative metrics of fitness that unify all theoretical and empirical subdisciplines in evolutionary biology and to translate theoretical concepts into quantities that can feasibly be measured in wild populations (Kozłowski 1993; de Jong 1994; Käär and Jokela 1998; Brommer 2000; Metcalf and Parvard 2007; Orr 2009; Hunt and Hodgson 2010; Sæther and Engen 2015; Grafen 2018). Perhaps the closest to broad conceptual unanimity is the idea that the “fittest” entities are ultimately those that contribute most descendants to a population at some point in the future (Benton and Grant 2000; Day and Otto 2001; Brommer et al., 2002, 2004; Hunt et al. 2004; Grafen 2006; Roff 2008; Hunt and Hodgson 2010; Graves and Weinreich 2017). Yet, such concepts can seem remote from the short‐term metrics of individual fitness that empiricists working on free‐living populations commonly aim to measure, which typically comprise simple functions of individuals’ realized survival and/or reproductive success (Brommer et al., 2002, 2004; Link et al. 2002; Coulson et al. 2006; Hendry et al. 2018; Wolak et al. 2018). Such metrics can correctly enumerate individual contributions to the next year or generation, but will not necessarily directly predict longer term genetic contributions, especially given density‐, frequency‐, and/or environment‐dependent selection (de Jong 1994; Day and Otto 2001; Hunt et al. 2004; Roff 2008; Sæther and Engen 2015; Graves and Weinreich 2017).

One opportunity to link these conceptual and empirical approaches stems from the principle of “individual reproductive value” (*V*_i_), here specifically defined as the number of copies of each of an individual's alleles that is expected to be present in future generations given the individual's realized pedigree of descendants (following Barton and Etheridge 2011). *V*_i_ results from Mendelian allele inheritance given the realized survival and reproductive success of a focal individual and successive generations of descendants, which in turn result from context‐dependent natural and sexual selection on multivariate expressed phenotypes alongside typically major components of environmental and demographic stochasticity (Sæther and Engen 2015; Snyder and Ellner 2018). In sexually reproducing species, any successful individual's ultimate genetic contribution will emerge over long timeframes (i.e., >>100 generations), but its expected genetic contribution (i.e., *V*_i_) should stabilize over relatively few generations: approximately log_2_(*N*), where *N* is population size (Chang 1999; Barton and Etheridge 2011). This equals approximately 7, 10, and 13 generations given *N* = 100, 1000, and 10,000, respectively, meaning that *V*_i_ should be estimable within timeframes that are increasingly within reach of empirical studies of free‐living populations, at least for species with moderately short generation times. Further, Barton and Etheridge (2011) showed that an individual's stabilized *V*_i_ accurately predicts the longer term (i.e., at generation *t*, where √(*N*log_e_(*N*))<<*t*<<*N*) probability that a neutral or weakly selected allele carried by that individual will persist in the focal population (versus go extinct), while the distribution of allele copy number conditional on persistence is independent of *V*_i_. Importantly, these results hold true even at the level of single individuals and alleles, not just across groups or classes of individuals. Consequently, an individual's stabilized *V*_i_ tightly predicts its longer term genetic contribution (Barton and Etheridge 2011).

These key theoretical results were initially derived assuming idealized conditions of neutral alleles segregating in a single well‐mixed (i.e., random mating, no population structure, or subdivision) diploid Wright–Fisher population with discrete nonoverlapping generations and constant population size that is sufficiently large to preclude short‐term inbreeding. However, they also apply adequately given some population structure and different distributions of reproductive success, and to alleles that affect reproductive success (given standard assumptions of the infinitesimal model, implying weak selection, Chang 1999; Barton and Etheridge 2011; Barton et al. 2017). To the degree that such short‐term stabilization and predictive ability of *V*_i_ hold under natural conditions encompassing age structure, overlapping generations, nonrandom mating, highly heterogeneous reproductive success within and between lineages, and dynamic finite population sizes with inbreeding and potentially nonzero immigration and emigration (Chang 1999; Gravel and Steel 2015), then the availability of multigeneration pedigree data from wild populations provides opportunities to directly quantify *V*_i_ and thereby infer longer term probabilities of allele persistence and individual genetic contributions. This provides a route to direct field quantification of what, to date, has been an abstract theoretical fitness concept.

Datasets that allow estimation of *V*_i_ can then be used to examine the degree to which more typically tractable short‐term metrics of individual fitness, comprising simple functions of individuals’ realized survival and reproductive success, can predict *V*_i_ and hence longer term individual genetic contributions. Such analyses facilitate further informed consideration of what evolutionary inferences can or cannot potentially be directly drawn from such metrics (Brommer et al., 2002, 2004). Metrics of individual lifetime fitness that are widely used by field biologists include lifetime reproductive success (LRS), a time‐independent metric defined as the total number of offspring produced by an individual over its lifetime, and individual growth rate (λ_ind_), a time‐dependent metric that emphasizes offspring produced early in life (McGraw and Caswell 1996; Käär and Jokela 1998; Brommer et al., 2002, 2004; MacColl and Hatchwell 2004, see Methods section). In principle, LRS and λ_ind_ can both predict individual long‐term genetic contributions given constant population size and lineage‐invariant selection, but λ_ind_ may be more appropriate in increasing populations (Brommer et al. 2002; Graves and Weinreich 2017). Both metrics are expected to out‐perform measures of individual survival or lifespan, especially given trade‐offs between survival and reproduction (Day and Otto 2001; Brommer et al. 2002; Hunt et al. 2004).

Yet, in reality, populations do not retain constant growth rates, but vary in size due to environmental stochasticity, and experience density‐, frequency‐, and/or environment‐dependent selection alongside substantial within‐lineage demographic stochasticity (de Jong 1994; Benton and Grant 2000; Hunt et al. 2004; Lande et al. 2009). Allele frequency dynamics then become very difficult to predict, especially in age‐structured populations, even if key rules governing selection and population dynamics are known (Day and Otto 2001; Lande et al. 2009; Gravel and Steel 2015; Sæther and Engen 2015; Myhre et al. 2016; Graves and Weinreich 2017). The degree to which metrics of individual lifetime fitness do (or do not) predict *V*_i_ and hence longer term individual genetic contributions given natural demographic variation and stochasticity, and thereby potentially provide any capability for direct long‐term evolutionary inference, then becomes an empirical question (Brommer et al., 2002, 2004; Hunt and Hodgson 2010; Graves and Weinreich 2017).

We use >20 years of complete genetically verified pedigree data from a free‐living song sparrow (*Melospiza melodia*) population to evaluate the degree to which individuals’ expected genetic contributions stabilize within an observed number of years and generations, thereby allowing individual *V*_i_ to be estimated and longer term probabilities of allele persistence and genetic contributions to be inferred. We then quantify the degree to which individuals’ stabilized *V*_i_ values are predicted by standard metrics of individual lifetime fitness (lifespan, LRS, and λ_ind_), and thereby elucidate what could potentially be directly inferred from such metrics in the context of natural environmental, demographic, genetic, and selective variation.

## Methods

### STUDY SYSTEM

Quantifying individual *V*_i_ (defined as the expected number of allele copies contributed to future generations conditional on an individual's realized pedigree of descendants, Barton and Etheridge 2011) requires complete, accurate, pedigree data spanning sufficient generations (approximately log_2_(*N*)) for the expectation to stabilize. This equates to approximately *G*log_2_(*N*) years, where *G* is mean generation time. Such multigeneration data exist for a small, largely philopatric, population of song sparrows inhabiting Mandarte island, Canada (full details in Supporting Information S1). The available data allow calculation of any desired metric of relatedness and fitness across individuals hatched since 1992, with little uncertainty or missing data with respect to the local population (Reid et al., 2014, 2016; Wolak et al. 2018, Supporting Information S1).

On Mandarte, among‐year variation in local environmental conditions and population density drives considerable among‐year variation in song sparrow reproduction and survival (Arcese et al. 1992; Wilson and Arcese 2003; Tarwater and Arcese 2018), inducing substantial among‐cohort variation in mean lifespan and LRS (Lebigre et al. 2012; Wolak et al. 2018). Total adult population size consequently varied substantially among years (arithmetic mean: 73 ± 29 SD individuals, range: 33–128, Supporting Information S1). The adult sex ratio was typically male biased (mean proportion males: 0.59 ± 0.07 SD, range: 0.39‐0.75, Supporting Information S1), allowing the mean and variance in LRS to differ between females and males (Lebigre et al. 2012). Generation time, calculated as mean parent age, was approximately 2.5 years (Supporting Information S2). Following basic theory (Chang 1999), individuals’ expected genetic contributions should therefore stabilize within roughly *G*log_2_(*N*) ≈ 2.5log_2_(73) ≈ 15 years, and potentially sooner given that the harmonic mean and effective population sizes are smaller than the arithmetic mean (Supporting Information S1). Stabilized *V*_i_ should therefore be estimable for song sparrows hatched early within the period for which genetically verified pedigree data for descendants are available (i.e., from 1992).

### CALCULATION OF INDIVIDUALS’ EXPECTED GENETIC CONTRIBUTIONS

The first objective was to calculate the number of copies of a (hypothetical) autosomal allele present in any focal individual that is expected to be present in the population in each year following the focal individual's natal year, and thereby evaluate each individual's stabilized *V*_i_. Such expectations can be readily calculated directly (i.e., analytically) from pedigree data using standard recursive algorithms for coefficients of kinship (Caballero and Toro 2000; Reid et al. 2016). However, the full distributions of allele copy numbers, and hence the variance around the expectation (stemming from multiple generations of Mendelian sampling) and associated probabilities of allele extinction, are not straightforward to calculate for individuals in complex pedigrees with irregular systems of inbreeding involving multiple ancestors (e.g., Hill and Weir 2011). We therefore used “gene‐dropping” simulations on the observed pedigree to compute key quantities (e.g., MacCluer et al. 1986; Caballero and Toro 2000, see below).

Because song sparrows have considerably overlapping generations (median age at first reproduction: 1 year; maximum lifespan: 10 years, Supporting Information S2), analyses focused on cohorts and years rather than discrete generations. Each individual hatched in a focal cohort that survived to adulthood (i.e., age one year) was assigned a unique allele identity, which was “dropped” down the observed pedigree assuming autosomal Mendelian inheritance (i.e., neutrally passed to each offspring of each sex with probability 0.5). The identities of all alleles present in all individuals in the total extant population (i.e., all adults and chicks, constituting a postbreeding census) in each subsequent year were extracted. Gene‐drops were replicated 8000 times. The mean number of copies of each allele present in each year (i.e., the expectation); the frequency of zero copies (and hence the probability of allele extinction, *P*_E_); and the mean, variance, and coefficient of variance (CV = standard deviation/mean) of allele copy number conditional on allele persistence (i.e., number of copies >0) were computed across replicates. Allele copies present in descendants of both sexes were counted irrespective of the sex of the focal individual in which an allele originated, thereby tracking allele propagation through the full pedigree rather than solely through single‐sex lineages (contra Brommer et al. 2004). Current simulations therefore assume that focal cohort individuals are heterozygous and unrelated at the hypothetical locus (i.e., single unique alleles), and correctly account for realized within‐lineage inbreeding in subsequent generations. They can therefore be considered to quantify the fate of a single neutral or weakly selected mutation in each focal individual (e.g., Barton and Etheridge 2011). Gene‐dropping was not applied to focal individuals that died before adulthood because their direct genetic contribution is known to be zero (hence *P*_E_ = 1), allowing them to be included in subsequent analyses without need for gene‐drop computations.

### ANALYSES OF INDIVIDUAL GENETIC CONTRIBUTIONS AND REPRODUCTIVE VALUE

Gene‐dropping focused on three song sparrow cohorts hatched during 1992–1994, for which ≥20 years of complete genetically verified pedigree data on descendants are in hand (Supporting Information S1). To evaluate whether individual stabilized *V*_i_ could be reliably ascertained, we examined whether individuals’ absolute or proportional expected genetic contributions to the total extant population in each year stabilized within an observed 20‐year posthatch timeframe. Proportional contributions were calculated by dividing each individual's absolute contribution (i.e., the expected number of allele copies) by the total number of alleles present in the population in the focal year (i.e., twice the total extant population size). This standardization facilitates direct comparison of values across years and cohorts given the varying population size. Stabilization was evaluated by calculating the Pearson correlation coefficient (*r*_p_) between individuals’ gene‐dropped expectations 20 years posthatch versus in each previous year, and the mean absolute difference (*μ*_d_) between individuals’ expectations between consecutive years. Convergence of *r*_p_ and *μ*_d_ toward one and zero, respectively, indicates stabilization.

We then quantified the degree to which an individual's expected genetic contribution 20 years posthatch (interpreted as its *V*_i_) predicts key attributes of the full distribution of allele copy number emerging across gene‐drop iterations. These analyses allow inspection of the degree to which empirical patterns concur with Barton and Etheridge's (2011) theoretical development, supporting the premise that *V*_i_ predicts an individual's longer term genetic contribution. First, to verify that *V*_i_ does tightly predict individual‐level probability of allele extinction within the observed 20‐year timeframe, and is consequently likely to do so across longer timeframes, we regressed the gene‐dropped probability of allele extinction (*P*_E_) after 20 years on *V*_i_ across individuals. Second, to quantify the degree to which *V*_i_ also predicts the full distribution of allele copy number conditional on allele persistence, we related the mean, variance, and CV of the number of copies after 20 years across gene‐drop iterations where the allele did not go extinct to *V*_i_.

### CALCULATION OF SHORT‐TERM METRICS OF INDIVIDUAL FITNESS

The second objective was to evaluate the degree to which standard short‐term metrics of individual realized lifetime fitness predict *V*_i_ and hence could in principle be used to directly infer individuals’ longer term genetic contributions. In the context of evolutionary quantitative genetic theory, lifetime fitness should ideally be measured zygote‐to‐zygote across a single generation (Lande and Arnold 1983; Wolf and Wade 2001; Hunt and Hodgson 2010). However, in practice, and in other contexts, fitness is commonly measured adult‐to‐offspring or adult‐to‐adult. This includes studies that aim to quantify fitness consequences of expression of adult traits (including reproductive or secondary sexual traits) and directly infer evolutionary outcomes, or to estimate *N*_e_ (e.g., Wolf and Wade 2001; Kokko et al. 2003; Hunt et al. 2004; MacColl and Hatchwell 2004; Sæther and Engen 2015; Myhre et al. 2016; Wolak et al. 2018). To encompass this spectrum of approaches, we extracted six lifetime fitness metrics for each focal individual. Lifespan was calculated as an individual's age in its last observed summer (one metric per individual). LRS was calculated as the total numbers of ringed (i.e., 6 days posthatch), independent (i.e., alive at cessation of parental care approximately 24 days posthatch) or recruited (i.e., age one year) genetic offspring produced by each focal individual over its lifetime (hence three metrics of LRS per individual). λ_ind_ was calculated as the dominant eigenvalue of an individual projection matrix of dimension equal to the individual's lifespan (McGraw and Caswell 1996; Brommer et al. 2002, Supporting Information S3). Top row fecundity terms were specified as either 0.5.m_ring_.ϕ_j_, where m_ring_ is the number of ringed offspring produced by each focal individual at each age and ϕ_j_ is the mean population‐wide juvenile survival rate in the focal year, or as 0.5.m_rec_, where m_rec_ is the number of recruited offspring produced by each focal individual at each age. This generates two metrics of λ_ind_ per individual, pertaining to ringed and recruited offspring, respectively (Supporting Information S3). The factor 0.5 accounts for the transmission probability of a focal parental allele to each offspring given Mendelian inheritance. This must be directly incorporated into the calculation of λ_ind_ prior to any further analyses, but can be readily applied as post hoc scaling factor for analyses of LRS (Brommer et al. 2004; Reid et al. 2016).

### ANALYSES OF INDIVIDUAL FITNESS AND REPRODUCTIVE VALUE

To evaluate the degree to which the six short‐term metrics of individual lifetime fitness explained and predicted variation in *V*_i_, we calculated Pearson and Spearman correlation coefficients, and linear regression slopes and associated adjusted *R* ^2^ values, between *V*_i_ and each fitness metric across individuals. These statistics were calculated using individuals’ absolute fitness and *V*_i_, and using relative fitness and *V*_i_ (i.e., individual value divided by the sex‐specific mean). Statistics were calculated across individuals from focal cohorts that survived to adulthood (as is currently common practice in studies that examine variation in fitness associated with adult phenotypes), and recalculated including values of zero for individuals that died before adulthood (thereby incorporating the otherwise “missing fraction”).

Because estimates of *V*_i_ for individuals hatched in 1992–1994 are partially nonindependent (because pedigrees are partially nested, Supporting Information S1), and because we considered multiple nonindependent fitness metrics, we report estimated correlation and regression parameters but do not focus on hypothesis testing. Regression intercepts were estimated for analyses of lifespan but forced through the origin otherwise, because individuals with zero LRS or λ_ind_ must have exactly zero *V*_i_. Because the total extant population sizes were similar across the three end years (i.e., 20 years posthatch for each cohort, Supporting Information S1), analyses of absolute and proportional *V*_i_ yielded similar conclusions (Supporting Information S4 and S5). Key results were also quantitatively unchanged if gene‐drops for all cohorts were run to the same end year (i.e., 22, 21, and 20 years posthatch for the 1992, 1993, and 1994 cohorts, respectively). Standard statistics (mean, variance, skew, CV) were used to summarize distributions of estimated *V*_i_ and fitness metrics. Analyses were implemented in R version 3.3.3 (R Core Team 2017) using package nadiv (Wolak 2012).

## Results

### INDIVIDUAL GENETIC CONTRIBUTIONS AND REPRODUCTIVE VALUE

**Figure 1:**
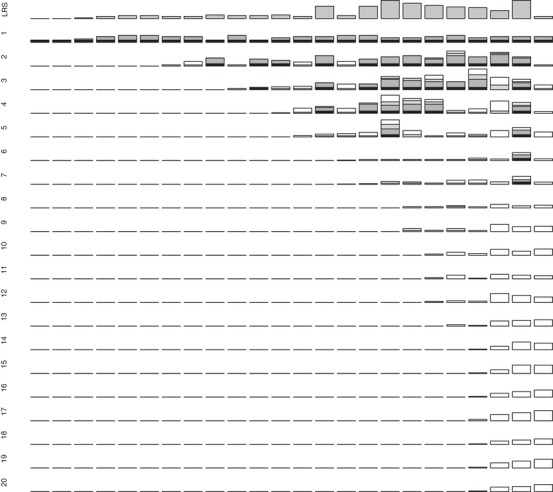
Observed lifetime reproductive success (LRS) measured as ringed offspring (top row) and absolute expected genetic contributions of 24 individual female song sparrows (columns) hatched in 1993 that survived to adulthood to the total extant population 1–20 years posthatch (descending rows). Black shading denotes genetic contributions arising because a focal female was still alive in the focal year. Dark gray, light gray, and white shading denote expected genetic contributions to offspring produced in the focal year, to surviving offspring produced in previous years, and to all subsequent descendants, respectively. All bars (except LRS) are scaled to maximum *y*‐axis values of eight allele copies to allow direct comparison across years. Columns (i.e., females) are ordered by increasing expected contributions across final observed years. Equivalent data for females hatched in 1992 and 1994, and proportional genetic contributions for all females, are shown in Supporting Information S4 and S5.

**Figure 2:**
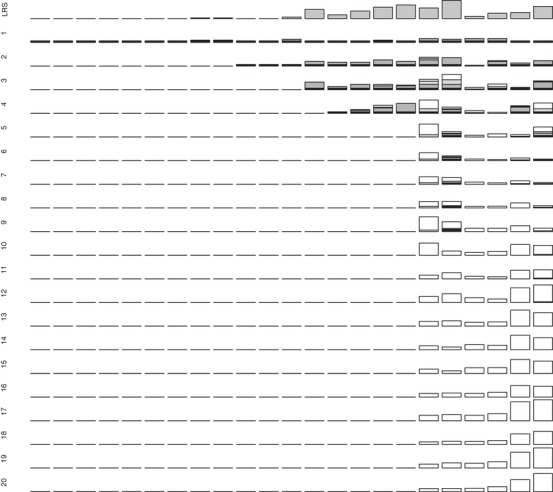
Observed lifetime reproductive success (LRS) measured as ringed offspring (top row) and absolute expected genetic contributions of 23 individual male song sparrows (columns) hatched in 1993 that survived to adulthood to the total extant population 1–20 years posthatch (descending rows). Figure attributes are as for Figure 1. All bars (except LRS) are scaled to maximum *y*‐axis values of 12 allele copies to allow direct comparison across years. Equivalent data for males hatched in 1992 and 1994, and proportional genetic contributions for all males, are shown in Supporting Information S4 and S5.

**Figure 3:**
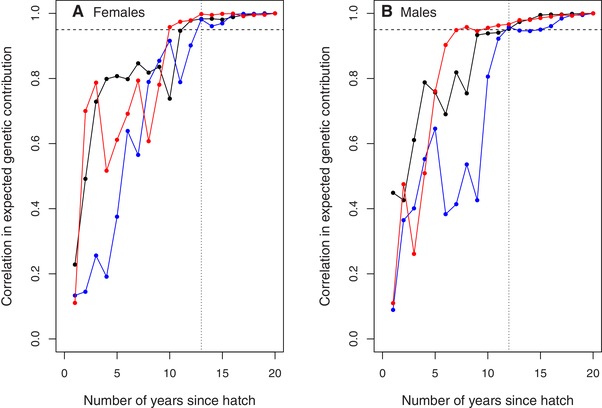
Pearson correlation coefficients between individuals’ absolute expected genetic contributions to the total extant population 20 years posthatch versus each previous year for adult (A) female and (B) male songs sparrows hatched in 1992 (black), 1993 (blue), and 1994 (red) that survived to adulthood. Dashed horizontal lines demarcate correlations that equal or exceed 0.95. Dotted vertical lines demarcate the numbers of years posthatch at which the correlation reached 0.95 for all three cohorts. Correlation coefficients were virtually identical given proportional rather than absolute expected genetic contributions.

In total, 21, 24, and 10 female and 38, 23, and 23 male song sparrows hatched in 1992, 1993, and 1994, respectively, survived to adulthood (i.e., age one year). These individuals’ absolute and proportional expected genetic contributions to the total extant population on Mandarte in each subsequent year clearly stabilized within the observed 20 year timeframe (Figs. 1 and 2; Supporting Information S4 and S5). Quantitatively, the correlations *r*_p_ between the genetic contributions expected after 20 years and in each preceding year exceeded 0.95 by 12–13 years posthatch in both sexes, and were typically close to one by the basic theoretical expectation of *∼*15 years posthatch (Fig. 3). Correspondingly, the mean deviations *μ*_d_ were very small (<0.0005, Supporting Information S5). This implies that the expectations evident by 20 years posthatch, and indeed after ∼13 years posthatch, can be interpreted as good approximations of individual *V*_i_ (as defined by Barton and Etheridge 2011).

**Figure 4:**
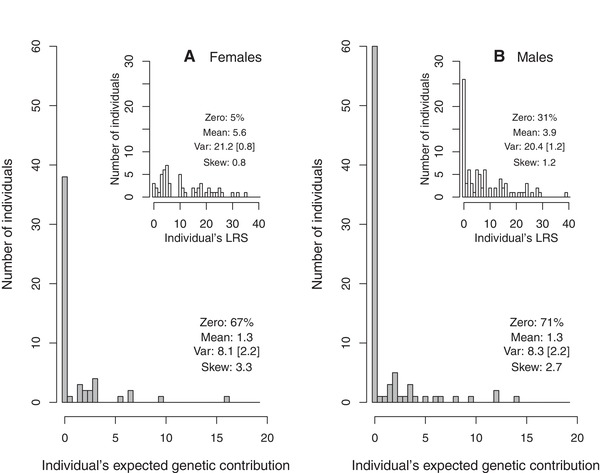
Distributions of individuals’ absolute expected genetic contributions to the total extant population 20 years posthatch (i.e., estimated reproductive value, *V*_i_) in (A) 55 female and (B) 84 male song sparrows hatched during 1992–1994 that survived to adulthood and these individuals’ lifetime reproductive success (LRS) measured as ringed offspring (inset panels). Y‐axis scales are standardized to facilitate comparison. Descriptive statistics comprise the percentage of individuals with values of zero, mean, variance (Var), skew, and coefficient of variance (CV, in square brackets). Statistics were calculated for 0.5LRS to facilitate direct comparison with *V*_i_. Corresponding statistics for full distributions including values of zero for individuals that died before adulthood are as follows: *V*_i_: mean 0.2 and 0.4, variance 1.7 and 2.8, skew 8.4 and 5.5, CV 5.6 and 4.7; and 0.5LRS: mean 1.0 and 1.2, variance 8.5 and 9.3, skew 3.3 and 3.1, CV 2.9 and 2.9, for females and males, respectively. Further summary statistics are in Supporting Information S7.

The distributions of individuals’ expected genetic contributions show that 67% of females and 71% of males that survived to adulthood made zero contribution to the total extant population 20 years later (i.e., *V*_i_ = 0, Fig. 4). This occurred even though many individuals had nonzero values for short‐term metrics of fitness (e.g., adult LRS measured as ringed offspring, Figs. 1, 2, and 4; Supporting Information S4). The sex‐specific distributions of *V*_i_ were consequently highly skewed; few individuals per cohort contributed to the genetic composition of subsequent generations (Fig. 4). The variance in *V*_i_ was smaller than the variance in 0.5LRS in both sexes, both across individuals that survived to adulthood and across all hatched individuals, but the CV was slightly larger for *V*_i_ (Fig. 4).

**Figure 5:**
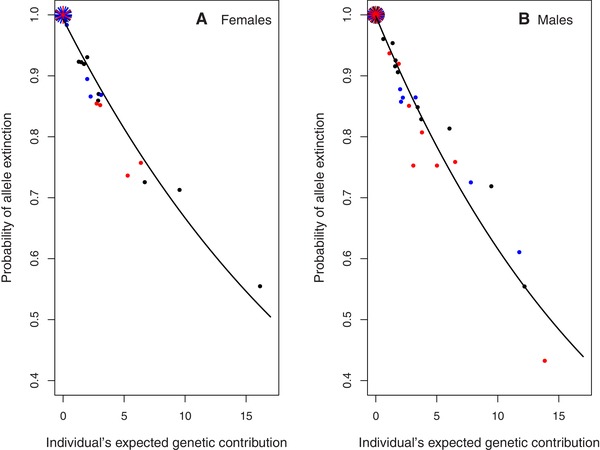
Relationships between an individual's probability of allele extinction (*P*_E_) and absolute expected genetic contribution to the total extant population 20 years posthatch (i.e., estimated reproductive value, *V*_i_) for (A) female and (B) male song sparrows hatched in 1992 (black), 1993 (blue), and 1994 (red) that survived to adulthood. Short lines denote multiple individuals with zero expected genetic contribution and hence *P*_E_ = 1. Solid lines depict the expected exponential relationship (i.e., *P*_E_ = exp(–α*V*_i_); Barton and Etheridge 2011) fitted to all three cohorts combined. Estimated coefficients α were –0.040 and –0.048 for females and males, respectively, for absolute *V*_i_, and –19.9 and –21.8, respectively, for proportional *V*_i_.

**Figure 6:**
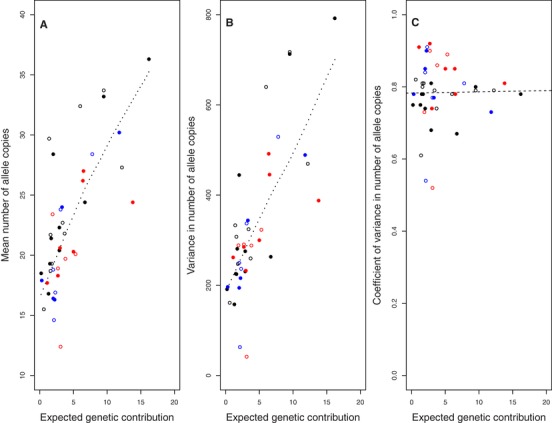
Relationships between an individual's absolute expected genetic contribution to the total extant population 20 years posthatch (i.e., estimated reproductive value, *V*_i_) and the (A) mean, (B) variance, and (C) coefficient of variance in the number of allele copies conditional on allele persistence (i.e., ≥1 copy) for 18 female (filled symbols) and 24 male (open symbols) song sparrows hatched in 1992 (black), 1993 (blue), and 1994 (red) with *V*_i_ > 0. In (A) and (B), dotted lines depict loess regressions (linear regressions were not fitted because estimated intercepts would not make biological sense). In (C), the dashed line denotes the linear regression (slope estimate: 0.001).

Individual *V*_i_ estimated 20 years posthatch tightly predicted the probability of allele extinction (*P*_E_) during the same timeframe (Fig. 5), as is expected and inevitable given the typically high *P*_E_ (Barton and Etheridge 2011). There was substantial variation in the number of allele copies present in the total extant population conditional on allele persistence (i.e., ≥1 copy), both among individuals (Fig. 6) and among gene‐drop iterations within individuals (Supporting Information S6). The mean and variance in copy number were both strongly positively associated with individual *V*_i_, but the CV was independent of *V*_i_ on average (Fig. 6). This concurs with the expectation that, conditional on allele persistence, the longer term distribution of genetic contributions will be independent of *V*_i_ (Barton and Etheridge 2011). The tight relationship between *V*_i_ and *P*_E_ that is already evident (Fig. 5) therefore implies that *V*_i_ encapsulates an individual's longer term genetic contribution.

### INDIVIDUAL FITNESS METRICS AND REPRODUCTIVE VALUE

**Figure 7:**
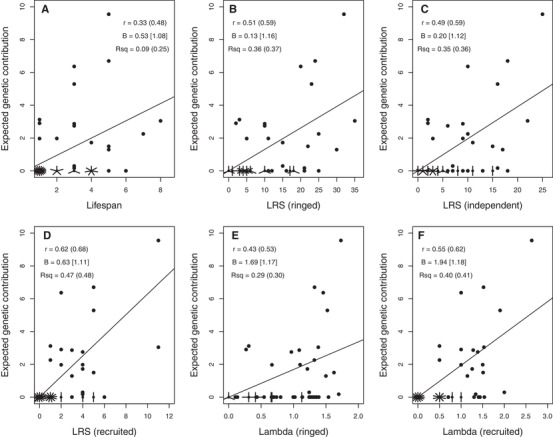
Relationships between an individual's absolute expected genetic contribution to the total extant population 20 years posthatch (i.e., estimated reproductive value, *V*_i_) and six short‐term metrics of fitness across 55 female song sparrows hatched in 1992–1994 that survived to adulthood. Fitness metrics are (A) lifespan, lifetime reproductive success (LRS) measured as (B) ringed, (C) independent, and (D) recruited offspring, and λ_ind_ (Lambda) measured across (E) ringed and (F) recruited offspring. Short lines denote multiple individuals with identical values. Solid lines denote linear regressions (forced through the origin in B–F). Statistics are Pearson correlation coefficient (*r*), linear regression slope (B), and adjusted *R* ^2^ (Rsq) calculated for absolute values of *V*_i_ and fitness across adults, or including individuals that died before adulthood (in parentheses), or using mean‐standardized relative values (in square brackets). Further summary statistics are in Supporting Information S7.

**Figure 8:**
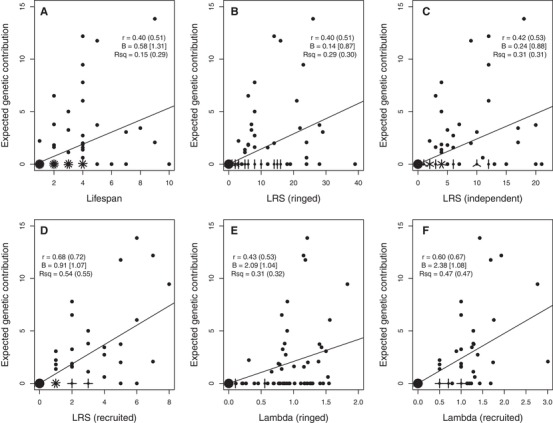
Relationships between an individual's absolute expected genetic contribution to the total extant population 20 years posthatch (i.e., estimated reproductive value, *V*_i_) and six short‐term metrics of fitness across 84 male song sparrows hatched in 1992–1994 that survived to adulthood. Fitness metrics are (A) lifespan, lifetime reproductive success (LRS) measured as (B) ringed, (C) independent, and (D) recruited offspring, and λ_ind_ (Lambda) measured across (E) ringed and (F) recruited offspring. Figure attributes are as for Figure 7. Further summary statistics are in Supporting Information S7.

Across the totals of 55 females and 84 males hatched during 1992–1994 that survived to adulthood, individual *V*_i_ estimated 20 years posthatch was positively associated with each of the six metrics of individual lifetime fitness (Figs. 7 and 8; Supporting Information S7). However, correlation coefficients were moderate for lifespan (∼0.3–0.4) and for metrics of ringed and independent offspring (∼0.4–0.5), and still far from unity for metrics of recruited offspring (∼0.6–0.7, Figs. 7 and 8; Supporting Information S7). As might be expected, there is considerable scatter around estimated linear regressions, meaning that the focal fitness metrics typically explain less than half the estimated among‐individual variation in *V*_i_ (Figs. 7 and 8; Supporting Information S7). Although the slopes of regressions of absolute *V*_i_ on absolute fitness metrics commonly diverged substantially from one, those for relative (i.e., mean‐standardized) *V*_i_ on relative fitness metrics were fairly close to one (Figs. 7 and 8; Supporting Information S7). This implies that metrics of individuals’ relative lifetime fitness can potentially provide unbiased predictors of relative genetic contributions to future generations on average, despite the considerable individual‐level deviation. There was no substantial difference in predictive ability between LRS and λ_ind_ measured to analogous offspring life stages (Figs. 7 and 8; Supporting Information S7). These conclusions, and particularly regression slopes and *R* ^2^ values, did not change markedly when including values of zero *V*_i_ and fitness for totals of 248 females and 194 males hatched during 1992–1994 that died before adulthood (Figs. 7 and 8; Supporting Information S7), or using proportional rather than absolute *V*_i_.

## Discussion

All attempts to explain and predict evolutionary dynamics require some assessment of the “fitness” of alleles and/or individual organisms; yet theoretical fitness concepts can be hard to reconcile with empirical realities encompassing overlapping generations, environmental and demographic stochasticity, and context‐dependent selection and genetic variation (de Jong 1994; Hunt and Hodgson 2010; Sæther and Engen 2015; Graves and Weinreich 2017). Relationships between short‐term metrics of individual fitness and phenotypic trait values define selection gradients, which can be combined with information on additive genetic (co)variances among traits of interest to infer evolutionary outcomes (conditional on fulfilling key assumptions of evolutionary quantitative genetic theory, Lande and Arnold 1983). Yet, especially in systems where genetic (co)variances cannot be readily quantified and/or major cross‐generation effects (e.g., maternal effects) are postulated, evolutionary outcomes are commonly discussed and inferred solely based on observed variation in individual or lineage fitness and associations with focal phenotypes (Kokko et al. 2003; Hunt et al. 2004; Hunt and Hodgson 2010). Any possible success of such purely phenotypic inferences depends, not least, on the degree to which available short‐term metrics of individual fitness do reliably predict longer term genetic contributions (Benton and Grant 2000; Brommer et al. 2002; Hunt et al. 2004). However, few studies have attempted to quantify such relationships in populations experiencing natural environmental, demographic, genetic, and selective variation. Brommer et al. (2004) used partial pedigree data from open populations of collared flycatchers (*Ficedula albicollis*) and ural owls (*Strix uralensis*) to relate individual LRS and λ_ind_ to longer term genetic contributions estimated over more than two generations. However, genetic contributions were only tracked through maternal lineages, meaning that persistence of autosomal alleles, which are propagated through both sexes, was probably greatly underestimated (Brommer et al. 2004). Individual LRS (measured as recruited offspring) and λ_ind_ explained only one‐third of observed variation in a number of locally fledged grand‐offspring (i.e., one further generation) in long‐tailed tits (*Aegithalos caudatus*, MacColl and Hatchwell 2004). Consequently, the general failure to measure individual “fitness” across multiple generations has even been highlighted as a shortcoming in the context of phenotypic approaches to testing sexual selection theory (Kokko et al. 2003; Hunt et al. 2004, but see Hunt and Hodgson 2010).

Our analyses of ≥20 years (i.e., approximately eight generations on average) of locally complete, accurate, pedigree data from free‐living song sparrows showed that individuals’ expected genetic contributions to the total extant population approximately stabilized within the observed timeframe (Figs. 1, 2, 3). “Individual reproductive value” (*V*_i_, as defined by Barton and Etheridge 2011) can consequently be estimated, and showed considerable among‐individual variation in both sexes (Fig. 4). As expected, *V*_i_ tightly predicted the short‐term probability of allele extinction (or, conversely, persistence), and is therefore likely to be highly quantitatively informative regarding longer term individual genetic contributions (Barton and Etheridge 2011). But, widely used metrics of individual lifetime fitness typically explained less than half the observed among‐individual variation in *V*_i_ (Figs. 7 and 8).

The availability of tractable metrics of observed phenotypic fitness that do partially predict individuals’ expected longer term genetic contributions in nature, despite the complexity and inevitable stochasticity of all underlying processes, could be deemed a success. However, there is unsurprisingly substantial unexplained variation, and hence potential to draw erroneous evolutionary inferences directly from purely phenotypic analyses of observed among‐individual variation in short‐term fitness. Discrepancies arose because some individuals with low (but non‐zero) LRS and λ_ind_ made substantial longer term genealogical and hence expected genetic contributions to the focal population, and because many individuals with non‐zero LRS and λ_ind_ made zero longer term contribution (Figs. 1, 2, and 4; Supporting Information S4). These outcomes do not simply reflect the focal population's small size. In sexually reproducing species with limited reproductive capacity (e.g., any species with substantial parental care), probabilities of lineage and allele extinction will be high irrespective of total population size, simply due to the inevitable substantial individual‐level environmental and demographic stochasticity and drift (e.g., Gravel and Steel 2015). However, allele extinction probabilities must also depend on mean demographic rates and population dynamics (e.g., Metcalf and Parvard 2007; Gravel and Steel 2015; Graves and Weinreich 2017). The focal song sparrow population decreased in size in 1998–1999 (Supporting Information S1), which was sufficiently soon after the focal cohorts hatched to potentially eliminate all descendants of some individuals. Such lineage extinctions become less likely across subsequent generations, because all individuals with nonzero expected genetic contributions will, in the medium term, be genealogical ancestors of all extant population members (Chang 1999; Caballero and Toro 2000; Barton and Etheridge 2011; but see Gravel and Steel 2015). However variation in local population size, caused by environmentally induced variation in fitness, will affect almost all populations and subpopulations in nature, and will consequently be integral to any evolutionary outcome (Sæther and Engen 2015; Engen and Sæther 2017). The focal song sparrow population is largely philopatric (Supporting Information S1), facilitating estimation of *V*_i_. In more dispersive populations, similar patterns to those observed in song sparrows are likely to hold across a larger spatial scale that encompasses dispersal, while individual *V*_i_ values measured more locally would typically tend to decrease toward zero.

An individual's observed LRS and λ_ind_ can be viewed as stochastic realizations of its underlying propensity for fitness, which is not directly observable on individuals as opposed to estimable from groups or classes of individuals (McGraw and Caswell 1996; Link et al. 2002; Snyder and Ellner 2018). Yet, realized lifetime fitness, comprising the number of offspring that was actually produced, might be envisaged as a reasonable predictor of longer term genetic contribution that captures the implications of initial individual‐level realizations of environmental and demographic stochasticity in survival and reproductive success (e.g., Sæther and Engen 2015). Predictive capability will also depend partly on the additive genetic variance and heritability in LRS, which is nonzero but small in song sparrows (<0.1 measured approximately chick‐to‐chick, implying that ∼90% of phenotypic variation represents “stochasticity”; Wolak et al. 2018). Such small or moderate values may be broadly typical, although still surprisingly few rigorous estimates are available (Shaw and Shaw 2014; Hendry et al. 2018). Overall, therefore, the song sparrow data serve to illustrate the degree to which measures of longer term genetic contributions are in practice removed from short‐term metrics of phenotypic fitness that are directly observable on individuals (e.g., de Jong 1994).

If direct estimation of individual *V*_i_ is, or soon could be, within reach of at least some field studies, what are its uses? *V*_i_ substantially provides the correct answer as to which individuals are expected to make longer term genetic contributions to any focal population (Barton and Etheridge 2011). Yet, as a single summary number, *V*_i_ directly integrates effects of selection and drift acting across multiple generations and does not itself provide direct insights into which processes or traits cause overall outcomes. Observed phenotypic trait values of interest could potentially be related to *V*_i_ rather than to short‐term fitness metrics, thereby directly capturing associations between phenotypes and longer term genetic contributions. However, as studies that can quantify individual *V*_i_ will necessarily have multigeneration pedigree (and/or genomic) data, such data might often be more insightfully deployed to estimate genetic covariances among traits of interest and single‐generation metrics of fitness and thereby employ the well‐established machinery of evolutionary quantitative genetics. In principle, and conditional on key assumptions, well‐specified quantitative genetic analyses can distinguish genetic (co)variances from environmental (co)variances, quantify evolutionary constraints and cross‐generational effects, and predict overall evolutionary outcomes (de Jong 1994; Morrissey et al. 2010; Reid 2012; Shaw and Shaw 2014). Yet, such approaches and their extrapolation to evolutionary predictions across multiple generations also face considerable challenges, especially given class structure, density‐, frequency‐, and environment‐dependent selection and genetic variation, and inbreeding and other interactions among relatives. Because individual *V*_i_ represents the outcome of genetic and environmental effects acting across multiple generations, it should not generally be directly treated as a focal trait in quantitative genetic analysis. Rather, further direct consideration of the structure of individual pedigrees and genealogies and hence *V*_i_ might provide useful complementary insights into evolutionary outcomes, including age‐ and sex‐structured contributions, lineage introgression, individuals responsible for inbreeding, and ultimately inclusive fitness (e.g., Caballero and Toro 2000; Suwanlee et al. 2007; Barton and Etheridge 2011; Newman and Easteal 2015).

Associate Editor: A. Charmantier

## Supporting information


**S1**. Study system, pedigree, population size and sex ratio.
**S2**. Generation time and adult ages.
**S3**. Metrics of individual fitness.
**S4**. Absolute expected genetic contributions for song sparrows hatched in 1992 and 1994.
**S5**. Proportional expected genetic contributions for song sparrows hatched in 1992‐1994.
**S6**. Distributions of allele frequencies.
**S7**. Additional summary statistics.
